# The 89-kDa PARP1 cleavage fragment serves as a cytoplasmic PAR carrier to induce AIF-mediated apoptosis

**DOI:** 10.1074/jbc.RA120.014479

**Published:** 2020-11-24

**Authors:** Masato Mashimo, Mayu Onishi, Arina Uno, Akari Tanimichi, Akari Nobeyama, Mana Mori, Sayaka Yamada, Shigeru Negi, Xiangning Bu, Jiro Kato, Joel Moss, Noriko Sanada, Ryoichi Kizu, Takeshi Fujii

**Affiliations:** 1 Department of Pharmacology, Faculty of Pharmaceutical Sciences, Doshisha Women's College of Liberal Arts, Kyotanabe, Kyoto, Japan; 2 Department of Clinical Pharmacy, Faculty of Pharmaceutical Sciences, Doshisha Women's College of Liberal Arts, Kyotanabe, Kyoto, Japan; 3 Pulmonary Branch, National Heart, Lung, and Blood Institute, National Institutes of Health, Bethesda, Maryland, USA

**Keywords:** apoptosis, apoptosis-inducing factor, caspase, cell death, parthanatos, poly(ADP-ribosyl)ation, poly(ADP-ribose) polymerase 1, AIF, apoptosis-inducing factor, GST, glutathione-*S*-transferase, NLS, nuclear localization signal, PAR, poly(ADP-ribose), PARP, poly(ADP-ribose) polymerase

## Abstract

Poly(ADP-ribose) polymerase 1 (PARP1) is a nuclear protein that is activated by binding to DNA lesions and catalyzes poly(ADP-ribosyl)ation of nuclear acceptor proteins, including PARP1 itself, to recruit DNA repair machinery to DNA lesions. When excessive DNA damage occurs, poly(ADP-ribose) (PAR) produced by PARP1 is translocated to the cytoplasm, changing the activity and localization of cytoplasmic proteins, *e.g.*, apoptosis-inducing factor (AIF), hexokinase, and resulting in cell death. This cascade, termed parthanatos, is a caspase-independent programmed cell death distinct from necrosis and apoptosis. In contrast, PARP1 is a substrate of activated caspases 3 and 7 in caspase-dependent apoptosis. Once cleaved, PARP1 loses its activity, thereby suppressing DNA repair. Caspase cleavage of PARP1 occurs within a nuclear localization signal near the DNA-binding domain, resulting in the formation of 24-kDa and 89-kDa fragments. In the present study, we found that caspase activation by staurosporine- and actinomycin D-induced PARP1 autopoly(ADP-ribosyl)ation and fragmentation, generating poly(ADP-ribosyl)ated 89-kDa and 24-kDa PARP1 fragments. The 89-kDa PARP1 fragments with covalently attached PAR polymers were translocated to the cytoplasm, whereas 24-kDa fragments remained associated with DNA lesions. In the cytoplasm, AIF binding to PAR attached to the 89-kDa PARP1 fragment facilitated its translocation to the nucleus. Thus, the 89-kDa PARP1 fragment is a PAR carrier to the cytoplasm, inducing AIF release from mitochondria. Elucidation of the caspase-mediated interaction between apoptosis and parthanatos pathways extend the current knowledge on mechanisms underlying programmed cell death and may lead to new therapeutic targets.

Poly(ADP-ribose) polymerase 1 (PARP1), in the presence of NAD^+^, catalyzes poly(ADP-ribosyl)ation, generating poly(ADP-ribose) (PAR) chains attached to acceptor proteins (1, 2). Activation of PARP1 catalytic activity occurs in response to DNA damage. Poly(ADP-ribosyl)ation of acceptor proteins modifies their activities, structure, and/or location, which affects diverse cellular functions, including DNA repair, gene expression, and cell death (1, 2, 3, 4). PARP1 is a 116-kDa protein, consisting of three domains: DNA-binding domain (N terminal), automodification domain (central), and catalytic domain (C terminal) (1). The DNA-binding domain recognizes DNA strand breaks, resulting in its dimerization and catalysis by the catalytic domain of transautomodification with PAR of the automodification domain (2, 5). PARP1 possesses a nuclear localization sequence (NLS) near the DNA-binding domain and a caspase-cleavage site between the DNA-binding domain and the automodification domain (3). PARP1 is initially responsible for DNA repair; the negative charge of PAR polymers covalently attached to PARP1 and histone loosen chromatin structure and recruit the scaffold protein XRCC1 and other DNA-remodeling enzymes (1, 2, 3). During caspase-dependent apoptosis, PARP1 is cleaved by caspases 3 and 7 at its caspase-cleavage site into 24-kDa and 89-kDa fragments (6, 7, 8). The 24-kDa PARP1 fragment contains the DNA-binding motif and the NLS, whereas the 89-kDa PARP1 fragment contains the automodification and catalytic domains. After PARP1 cleavage by caspase, the 24-kDa PARP1 fragment irreversibly binds to DNA breaks and acts as a transdominant inhibitor of active PARP1, whereas the 89-kDa PARP1 fragment is translocated to the cytoplasm (9, 10). Thereby, PARP1 fragmentation by caspase leads to its inactivation, which inhibits DNA repair and facilitates caspase-mediated DNA fragmentation in apoptosis.

Parthanatos, a programmed cell death, is initiated by PARP1 over-reaction to DNA damage, which is seen in neurons in Parkinson's disease, after glutamate excitotoxicity and in brain ischemia (11, 12, 13). Suppression of parthanatos through PARP1 inhibition may have therapeutic potential in these diseases (14). On binding single- and double-stranded DNA breaks, activated PARP1 catalyzes the covalent addition of long and branched polymers of ADP-ribose to nuclear acceptor proteins, including PARP1 itself, XRCC1, and histones (1, 2). PAR translocation from the nucleus to the cytoplasm is a crucial step in the parthanatos pathway. After DNA damage, 90% of PAR polymers are synthesized by PARP1, and most PAR polymers are attached to PARP1 itself (1). Poly(ADP-ribose) glycohydrolase, a primary enzyme for PAR degradation, is involved in PAR translocation from the nucleus to cytoplasm (15, 16, 17). Poly(ADP-ribose) glycohydrolase endoglycosidase activity generates protein-free and small PAR polymers that appear to pass thorough nuclear membranes (15, 17, 18). After PAR polymers produced by PARP1 are translocated from the nucleus to the cytoplasm, they bind to apoptosis-inducing factor (AIF), which is anchored to the mitochondrial membrane (15, 19). PAR binding to AIF results in its release to the cytoplasm (13). As AIF has an NLS near its N terminus, released AIF is translocated to the nucleus and associates with DNAase, resulting in large-scale DNA fragmentation (20). PAR also interacts with hexokinase 1, which is the first enzyme in the glycolytic pathway, and inhibits its activity, leading to energy depletion (21). These pathways, including PAR synthesis and its translocation to induce parthanatos, are caspase independent (22).

To the contrary, AIF release and its translocation to the cytoplasm during apoptosis also has been demonstrated in response to several stimuli in diverse cell types (23). Proapoptotic Bcl-2 members, such as Bax and Bak, induce a selective process of outer membrane permeabilization through the formation of channels, which allows AIF release from mitochondria (24). Calpain, a Ca^2+^-activated protease, releases AIF from mitochondria by cleaving membrane-bound AIF, which then induces AIF-mediated DNA fragmentation (25).

In this study, we report a novel route for AIF release during apoptosis. Caspase-3 activation after exposure to staurosporine and actinomycin D, conventional apoptosis inducers, resulted in PARP1-mediated PAR production and then PARP1 cleavage into 89-kDa and 24-kDa PARP1 fragments. The 89-kDa PARP1 fragments, with attached PAR polymers, were translocated from the nucleus to the cytoplasm, whereas 24-kDa fragments were associated with DNA breaks. The 89-kDa PARP1 fragments in the cytoplasm interacted with AIF via their PAR polymers, leading to AIF release and translocation to the nucleus, resulting in nuclear shrinkage. Thus, the 89-kDa PARP1 fragment generated by caspase-3 acts as a PAR carrier from the nucleus to the cytoplasm to induce AIF-mediated DNA fragmentation in caspase-mediated apoptosis.

## Results

### PARP1-mediated AIF release occurs after caspase activation

**Figure 1:**
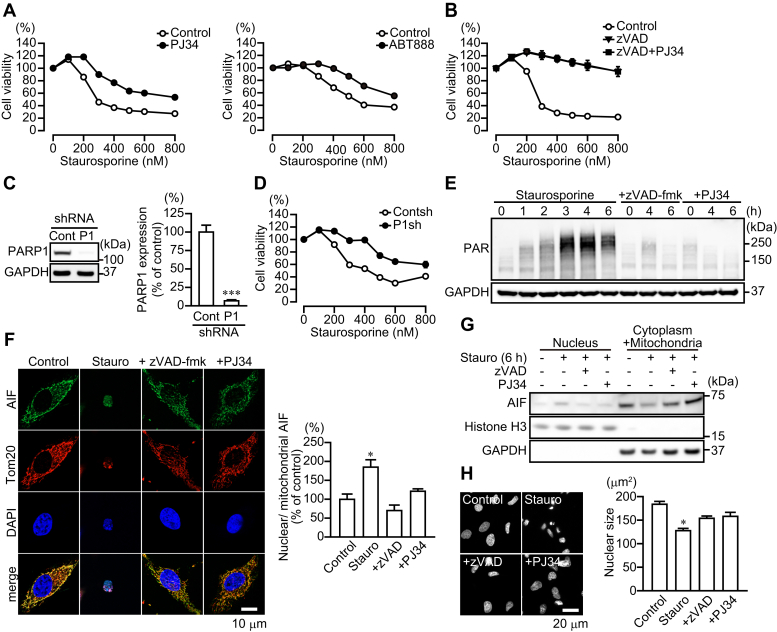
**PARP1-mediated AIF release after caspase activation after staurosporine exposure**. *A,* PARP-dependent cell death after staurosporine exposure. HeLa cells were exposed to staurosporine (6 h) at indicated concentrations. PJ34 (10 μM) (*left*) or ABT888 (10 μM) (*right*) was added for 30 min before staurosporine exposure (means ± S.E.M., n = 3). ∗∗∗*p* < 0.001 *versus* control at above 300 nM (two-way ANOVA with post hoc Tukey test). *B,* effect of zVAD-fmk on staurosporine-induced cell death. zVAD-fmk (50 μM) and PJ34 (10 μM) were added for 30 min before staurosporine exposure (means ± S.E.M., n = 3). ∗∗∗*p* < 0.001 *versus* control at above 200 nM (two-way ANOVA with post hoc Tukey test). *C,* PARP1 expression in HeLa cells stably expressing PARP1 shRNA. Right graph shows PARP1 expression levels. PARP1 protein level was normalized to GAPDH (means ± S.E.M., n = 3). ∗∗∗*p* < 0.001 *versus* control (Student's *t* test). *D,* effect of PARP1 depletion on cell viability after staurosporine exposure. Cells were exposed to staurosporine (6 h) at indicated concentrations (means ± S.E.M., n = 3). ∗∗∗*p* < 0.001 *versus* control at above 200 nM (two-way ANOVA with post hoc Tukey test). *E,* PAR production after staurosporine exposure. zVAD-fmk (50 μM) or PJ34 (10 μM) was added to the media for 30 min before 300 nM staurosporine exposure. GAPDH was used as the loading control. The bar graph represents PAR production assessed with pooled densitometric data (means ± S.E.M., n = 3). Data are normalized to the ratio of PAR to GAPDH at 0 h as 100%. *F,* AIF accumulation in nuclei of HeLa cells after 6-h exposure to 300 nM staurosporine. Cells were subjected to immunocytochemistry using anti-AIF antibody (*green*), anti-Tom20 antibody (*red*), and DAPI staining (*blue*). Right graph shows the ratio of nuclear to mitochondrial AIF fluorescence (means ± S.E.M., n = 3). ∗*p* < 0.05 *versus* control (one-way ANOVA with post hoc Tukey test). The scale bar represents 20 μm. *G,* AIF localization in nuclei. After 6-h exposure to staurosporine (300 nM), nuclear and cytoplasmic/mitochondrial fractions were separated. zVAD-fmk (50 μM) or PJ34 (10 μM) was added for 30 min before staurosporine exposure. Histone H3 and GAPDH were used as nuclear and cytoplasmic markers, respectively. *H,* nuclear shrinkage after 6-h exposure to 300 nM staurosporine. DAPI was used to measure nuclear sizes in HeLa cells. The scale bar represents 10 μm. Right graph shows mean nuclear sizes (means ± S.E.M., n = 100–300 cells). ∗*p* < 0.05 *versus* control (one-way ANOVA with post hoc Tukey test). AIF, apoptosis-inducing factor; DAPI, 4',6-diamidino-2-phenylindole; PARP1, poly(ADP-ribose) polymerase 1.

We investigated whether staurosporine, an apoptosis inducer, stimulated PARP1-dependent cell death. Exposure of HeLa cells to staurosporine for 6 h resulted in cytotoxicity (Fig. 1*A*). Pharmacological inhibition of PARP by PJ34 and ABT888 increased significantly the number of viable cells, whereas inhibition of caspase by zVAD-fmk suppressed cell death completely (Fig. 1*A*). PJ34 did not augment the improved survival rate seen with zVAD-fmk (Fig. 1*B*). In contrast to the effects of PARP1 inhibition by PJ34, pharmacological inhibition of tankyrase (PARP5) by XAV939 did not alter the sensitivity to staurosporine-induced cytotoxicity to the extent seen with PJ34 (Fig. S1*A*). Stable expression of PARP1 shRNA reduced PARP1 protein to 10% of that in HeLa cells transfected with control shRNA (Fig. 1*C*). HeLa cells expressing PARP1 shRNA showed reduced staurosporine-induced cytotoxicity, compared with control shRNA, similar to that seen with PJ34 (Fig. 1*D*). In addition, pretreatment with PJ34 did not improve the viability of Hela cells expressing PARP1 shRNA (Fig. S1*B*), suggesting that PJ34 has specificity to PARP1-dependent cell death induced by staurosporine. Exposure to N-methyl-N'-nitro-N-nitrosoguanidine, a DNA-alkylating agent, resulted in PARP1-dependent and caspase-independent cell death, because PJ34, but not zVAD-fmk, reduced N-methyl-N'-nitro-N-nitrosoguanidine-induced cytotoxicity (Fig. S1*C*). PARP1 activation results in PAR synthesis and then AIF release from mitochondria (22). Western blot analysis revealed that PAR was generated as early as 1 h after exposure to staurosporine, and approached the peak at 4 h, with increased PAR lasting at least past 6 h (Fig. 1*E*). After exposure to staurosporine for 6 h, AIF accumulated in HeLa cell nuclei, leading to nuclear shrinkage (Fig. 1, *F*–*H*). The pharmacological inhibition of either caspase or PARP1 prevented PAR synthesis as well as AIF-mediated nuclear shrinkage (Fig. 1, *E*–*H*). Moreover, HeLa cells expressing PARP1 shRNA did not exhibit PAR synthesis, AIF translocation to nuclei, and nuclear shrinkage (Fig. S2, *A*–*C*). All findings are consistent with the notion that PARP1 activation and then AIF release from mitochondria appears to be partially required downstream of caspase-mediated apoptosis after staurosporine exposure.

### 89-kDa PARP1 fragments are translocated from the nucleus to the cytoplasm after exposure to staurosporine

**Figure 2:**
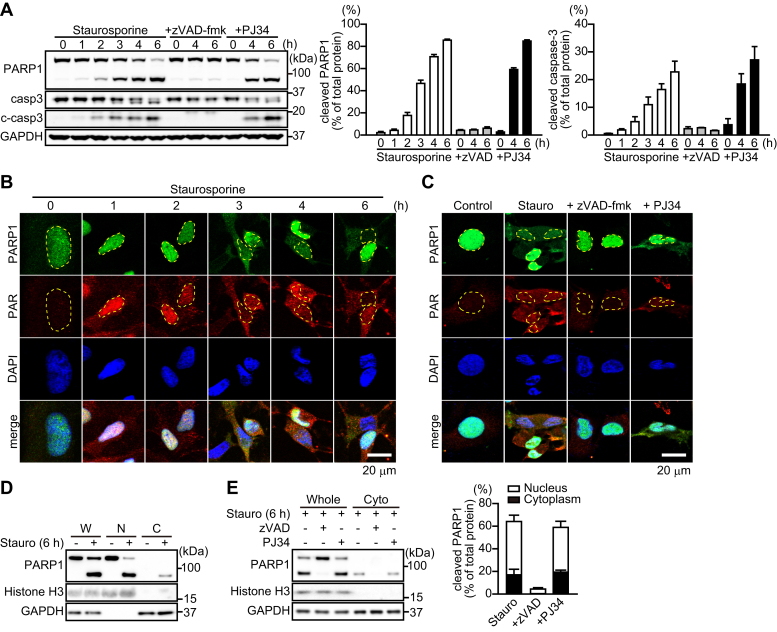
**PARP1 cleavage by caspase-3 results in the translocation of the 89-kDa PARP1 fragment to the cytoplasm.***A,* cleavage of PARP1 and caspase-3 (c-caspase-3) after staurosporine exposure. zVAD-fmk (50 μM) or PJ34 (10 μM) was added to the media for 30 min before 300 nM staurosporine exposure. Anti-PARP1 antibody recognizes full-length PARP1 and its 89-kDa PARP1 fragment. GAPDH was used as the loading control. The bar graphs represent cleaved PARP1 (*left*) and caspase-3 (*right*), respectively, assessed with pooled densitometric data (means ± S.E.M., n =3). Data are normalized to the ratio of cleaved proteins to total proteins. *B,* time-dependent change in the localization of PARP1 and PAR after 300 nM staurosporine exposure for indicated times. Cells were subjected to immunocytochemistry using anti-PARP1 antibody (*green*), anti-PAR antibody (*red*), and DAPI staining (*blue*). Yellow lines indicate the position of nuclei. The scale bar represents 20 μm. *C,* effect of caspase and PARP inhibition on PARP1 localization. zVAD-fmk (50 μM) or PJ34 (10 μM) was added for 30 min before 6-h exposure to 300 nM staurosporine. The scale bar represents 20 μm. *D,* subcellular localization of PARP1 after 6-h exposure to staurosporine (300 nM). Subcellular fractionation was performed to separate nuclei (N) and cytoplasmic (C) fractions. Histone H3 and GAPDH were used as nuclear and cytoplasmic markers, respectively. W indicates whole cells. *E,* effect of caspase and PARP inhibition on subcellular PARP1 localization. zVAD-fmk (50 μM) or PJ34 (10 μM) was added for 30 min before a 6-h exposure to staurosporine (300 nM). The bar graph represents the ratio of cleaved PARP1 to total PARP1 in nuclear (*white*) and cytoplasmic (*black*) fractions, assessed with pooled densitometric data (means ± S.E.M., n = 3). DAPI, 4',6-diamidino-2-phenylindole; PAR, poly(ADP-ribose); PARP1, poly(ADP-ribose) polymerase 1.

PARP1 is proteolytically cleaved near the third zinc-finger domain by caspases-3 and 7 into 24-kDa N-terminal and 89-kDa C-terminal PARP1 fragments (6). Western blot analysis using the PARP1 antibody, which recognizes full-length PARP1 and its 89-kDa PARP1 fragments, detected 89-kDa PARP1 fragments after a 1-h exposure to staurosporine, which resulted in caspase-3 activation (Fig. 2*A*). zVAD-fmk, but not PJ34, inhibited caspase-3 activation as well as PARP1 fragmentation after 4 and 6 h of staurosporine exposure (Fig. 2*A*). PARP1 knockdown by shRNA did not affect caspase-3 activation (Fig. S2*D*).

Using immunocytochemistry to evaluate intracellular localization of PARP1 and PAR, we found that, after exposure to staurosporine, PARP1 was translocated from the nucleus to the cytoplasm after 3 h; in contrast, PAR was located primarily in the nuclei of HeLa cells at 1 h and translocated to the cytoplasm after 3 h (Fig. 2*B*). zVAD-fmk and PJ34 reduced cytoplasmic PAR content after 6-h exposure to staurosporine, whereas zVAD-fmk, but not PJ34, inhibited the cytoplasmic localization of PARP1 (Fig. 2*C*). Moreover, HeLa cells expressing PARP1 shRNA exhibited reduced translocation of PAR to the cytoplasm after 6-h exposure to staurosporine compared with control shRNA (Fig. S2*E*).

We next investigated the molecular state of PARP1. Subcellular fractionation confirmed that, after 6-h exposure to staurosporine, only the 89-kDa PARP1 fragment, but not full-length PARP1, was localized in the cytoplasm (Fig. 2*D*). zVAD-fmk, but not PJ34, prevented both PARP1 fragmentation and the cytoplasmic localization of 89-kDa PARP1 fragments (Fig. 2*E*). Densitometric analysis indicates that approximately 20% of PARP1 was present as the cleaved form in the cytoplasm (Fig. 2*E*). These results indicate that the 89-kDa PARP1 fragment is translocated to the cytoplasm after PARP1-mediated PAR production in nuclei.

**Figure 3:**
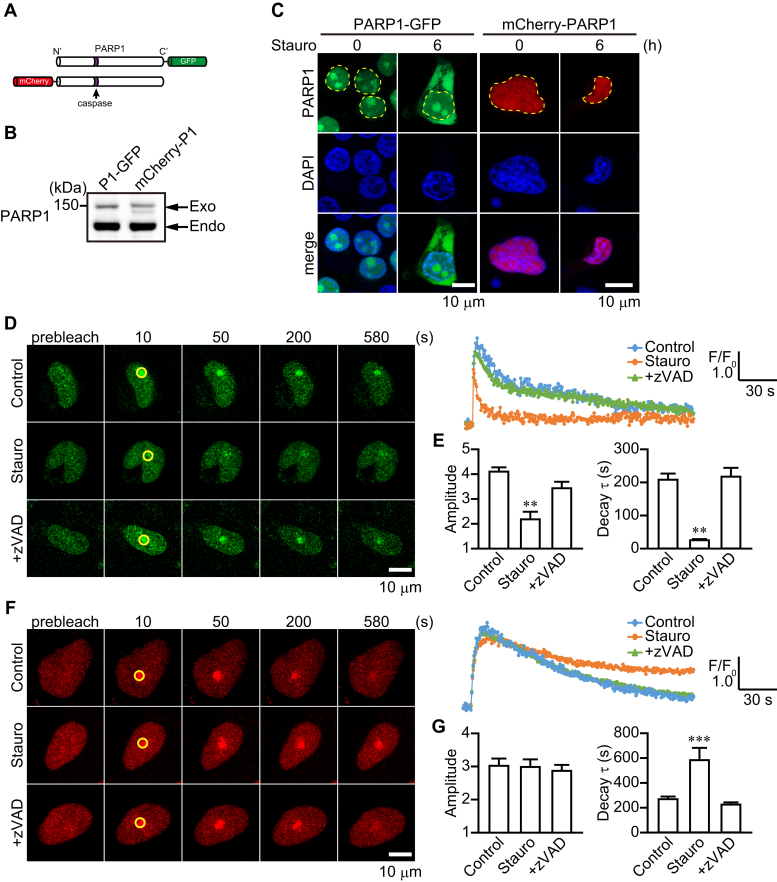
**Different dynamics of PARP1-GFP and mCherry-PARP1 after staurosporine exposure.***A,* schematic diagram of PARP1-GFP and mCherry-PARP1 constructs. *B,* PARP1-GFP and mCherry-PARP1 expression in HeLa cells. After 1-day transfection, Western blotting using anti-PARP1 antibody was performed to confirm expression. The lower arrow indicates endogenous PARP1 expression in HeLa cells, whereas the upper arrow shows exogenous PARP1 expression. *C,* localization of PARP1 constructs after 6-h exposure to staurosporine (300 nM). Nuclei were stained with DAPI (*blue*). *Yellow* lines indicate the position of nuclei. The scale bar represents 10 μm. *D* and *F*, effect of caspase activation on the recruitment of PARP1 constructs to DNA lesions induced by microirradiation. zVAD-fmk (50 μM) was added for 30 min before 2-h exposure to staurosporine (300 nM). Cells transiently transfected with PARP1 constructs were subjected to microirradiation at the indicated circle. The scale bar represents 10 μm. Right graphs indicate the time course of fluorescence of PARP1 constructs at circles. Images were taken every 2 s for 10 min. *E* and *G,* effect of caspase activation on the mean amplitude (*left*) and decay time constant (*right*) of PARP1 constructs after microirradiation without or with staurosporine (300 nM) and zVAD-fmk (50 μM) (means ± S.E.M., n = 8). ∗∗*p* < 0.01, ∗∗∗*p* < 0.001 *versus* control (one-way ANOVA with post hoc Tukey test). DAPI, 4',6-diamidino-2-phenylindole; PARP1, poly(ADP-ribose) polymerase 1.

To investigate the subcellular localization of 89-kDa and 24-kDa PARP1 fragments after exposure to staurosporine, GFP and mCherry were linked to the C-terminal and N-terminal sites, respectively, of PARP1, generating PARP1-GFP and mCherry-PARP1, respectively (Fig. 3, *A*–*B*). After 6-h exposure to staurosporine, the PARP1-GFP signal was localized in the cytoplasm and nucleus, whereas mCherry-PARP1 signal was confined to the nucleus (Fig. 3*C*). All findings are consistent with the notion that 89-kDa PARP1 fragments, but not 24-kDa PARP1 fragments, are translocated to the cytoplasm after PARP1 cleavage by caspase.

The recruitment of PARP1 at DNA lesions is required for PAR synthesis (26). We next investigated whether PARP1 is cleaved after it is recruited to DNA lesions. To address this issue, we performed a 405-nm laser microirradiation to induce DNA lesions in HeLa cells expressing PARP1 constructs. After microirradiation, PARP1-GFP and mCherry-PARP1 rapidly accumulated at DNA-damage sites and gradually disappeared (Fig. 3, *D*–*G*). Short-term exposure (2 h) to staurosporine suppressed PARP1-GFP recruitment and accelerated dissociation at DNA lesions, whereas zVAD-fmk blocked the effect (Fig. 3, *D*–*E*). In contrast, mCherry-PARP1 accumulated at DNA lesions in the presence of staurosporine for a longer time than occurred in the absence of staurosporine, whereas staurosporine exposure did not affect its accumulation level (Fig. 3, *F*–*G*). The prolonged accumulation of mCherry-PARP1 was blocked by the addition of zVAD-fmk to the extent seen with control (Fig. 3, *F*–*G*). These results suggest that caspases can cleave PARP1 that is recruited at DNA lesions and, after fragmentation, 89-kDa PARP1 fragments dissociate, whereas 24-kDa PARP1 fragments remain at the DNA lesions.

### The 89-kDa PARP1 fragment is poly(ADP-ribosyl)ated in the nucleus and translocated to the cytoplasm after exposure to staurosporine

**Figure 4:**
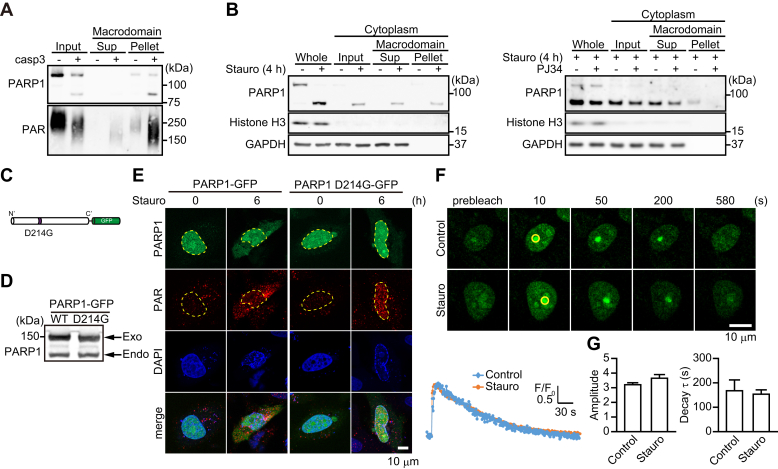
**89-kDa PARP1 fragments were translocated to the cytoplasm with attached PAR.***A,* GST pull-down assay using GST macrodomain. Recombinant human PARP1 proteins automodified by PAR were incubated with recombinant human caspase-3 proteins for 4 h. GST pull-down assay using GST macrodomain was used to isolate poly(ADP-ribosyl)ated proteins. *B,* poly(ADP-ribosyl)ated 89-kDa PARP1 fragments in the cytoplasm. HeLa cells were exposed to 300 nM staurosporine for 4 h (*left*) without or with PJ34 (10 μM) (*right*). After subcellular fractionation, cytoplasmic fractions were subjected to GST pull-down assay using GST macrodomain. Histone H3 and GAPDH were used as nuclear and cytoplasmic markers, respectively. *C,* schematic diagram of PARP1 D214G-GFP construct. *D,* PARP1-GFP WT and D214G expression in HeLa cells. After 1-day transfection, Western blotting using anti-PARP1 antibody was performed to confirm expression. The lower arrow indicates endogenous PARP1 expression in HeLa cells, whereas the upper arrow shows exogenous PARP1 expression. *E,* localization of PARP1 D214G-GFP and PAR after 6-h exposure to staurosporine (300 nM). Nuclei were stained with DAPI (*blue*). Yellow lines indicate the position of nuclei. The scale bar represents 10 μm. *F,* effect of caspase activation on the recruitment of PARP1 D214G to DNA lesions induced by microirradiation with 2-h exposure to staurosporine (300 nM). Cells transiently transfected with PARP1 D214G-GFP were subjected to microirradiation without or with 300 nM staurosporine at the indicated circle. The scale bar represents 10 μm. Right graphs indicate the time course of fluorescence of PARP1 D214G at circles. Images were taken every 2 s for 10 min. *G,* effect of caspase activation on the mean amplitude (*left*) and decay time constant (*right*) of PARP1 D214G-GFP after microirradiation without or with 300 nM staurosporine (means ± S.E.M., n = 8) *versus* control (Student's *t* test). DAPI, 4',6-diamidino-2-phenylindole; PAR, poly(ADP-ribose); PARP1, poly(ADP-ribose) polymerase 1; WT, wild-type.

Based on the facts that caspase can cleave PARP1 after it is recruited to DNA lesions and that the 89-kDa PARP1 fragment possesses the automodification domain, we hypothesized that 89-kDa PARP1 fragments were translocated to the cytoplasm with attached PAR polymers. To address this issue, we performed a pull-down assay using a glutathione-*S*-transferase (GST)–tagged macrodomain immobilized on Glutathione Sepharose. The macrodomain is a PAR-binding motif in Histone H2A1.1. GST macrodomain specifically binds ADP-ribosylated proteins, which can then be isolated in a pull-down assay. Recombinant PARP1 proteins automodified by PAR polymers were incubated with recombinant capsase-3 proteins for 1 h. Pull-down assay using the GST macrodomain detected recombinant full-length PARP1 and 89-kDa PARP1 fragments (Fig. 4*A*). PAR polymers with attached 89-kDa PARP1 fragments were shifted to a lower molecular weight range than the full-length PARP1 (Fig. 4*A*). These results suggest that 89-kDa PARP1 fragments generated by caspase-3 still retained PAR polymers. In HeLa cells, after 4-h exposure to staurosporine, 89-kDa PARP1 fragments present in the cytoplasm were poly(ADP-ribosyl)ated (Fig. 4*B*, *left*). PJ34 did not suppress the generation of 89-kDa PARP1 fragments and its translocation to the cytoplasm but inhibited formation of poly(ADP-ribosyl)ated 89-kDa PARP1 fragments (Fig. 4*B*, *right*).

To confirm whether 89-kDa PARP1 fragments generated after staurosporine exposure carry PAR polymers from the nucleus to the cytoplasm, we prepared a PARP1 D214G-GFP construct, which is a mutant lacking the caspase-cleavage site (Fig. 4, *C*–*D*). In PARP1 shRNA-expressing HeLa cells, staurosporine exposure did not result in the translocation of PARP1 D214G-GFP and PAR to the cytoplasm (Fig. 4*E*). Moreover, after microirradiation, PARP1 D214G-GFP rapidly accumulated at DNA-damage sites and gradually disappeared regardless of the presence of staurosporine (Fig. 4, *F*–*G*). These results are consistent with the fact that generation of the 89-kDa PARP1 fragment by caspase cleavage is required for PAR translocation to the cytoplasm.

### The 89-kDa PARP1 fragment binds AIF via PAR polymers in the cytoplasm

**Figure 5:**
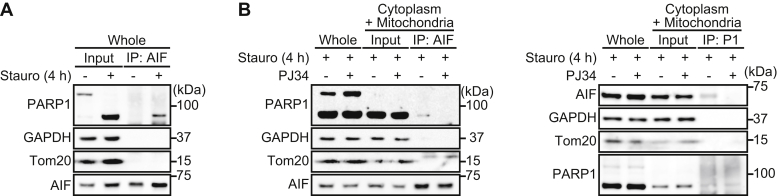
**The 89-kDa PARP1 fragment binds AIF via PAR polymers.***A,* the 89-kDa PARP1 fragment binds AIF. After 4-h exposure to staurosporine, cells were subjected to immunoprecipitation with anti-AIF antibody and then Western blotting using anti-PARP1 antibody. GAPDH was used for a loading control. *B,* 89-kDa PARP1 fragments bind AIF via PAR polymers in the cytoplasm. PJ34 (10 μM) was added for 30 min before 4-h exposure to staurosporine (300 nM). After subcellular fractionation, cytoplasmic/mitochondrial fractions were subjected to immunoprecipitation with anti-AIF antibody and anti-PARP1 antibody and then Western blotting using anti-PARP1 antibody (*left*) and anti-AIF antibody (*right*), respectively. GAPDH and Tom20 were used as cytoplasmic and mitochondrial markers, respectively. AIF, apoptosis-inducing factor; IP, immunoprecipitation; PARP1, poly(ADP-ribose) polymerase 1.

In the cytoplasm, PAR binding to AIF is a critical step involved in the release of AIF from mitochondrial membranes (19). We next investigated whether 89-kDa PARP1 fragments interact with AIF via PAR polymers after translocation to the cytoplasm. After 4-h exposure to staurosporine, the 89-kDa PARP1 fragments, but not the full-length PARP1, interacted with AIF (Fig. 5*A*). Moreover, in cytoplasm, the 89-kDa PARP1 fragments interacted with AIF, which was suppressed by PJ34 (Fig. 5*B*, *left*). Reciprocal immunoprecipitation with anti-AIF antibody confirmed that 89-kDa–cleaved PARP1 fragments interacted with AIF in cytoplasmic and mitochondrial fractions after a 4-h exposure to staurosporine (Fig. 5*B*, *right*). These results indicate that the 89-kDa PARP1 fragments interacted with AIF in the cytoplasm via PAR polymers.

### Transautomodification of PARP1 by PAR and its fragmentation by caspase are required for AIF binding

PARP1 E988K mutant has a mutation in the catalytic domain and loses the ability to catalyze poly(ADP-ribosyl)ation (Fig. S3*A*). In PARP1 shRNA-expressing HeLa cells, expression of PARP1 E988K-GFP did not produce PAR after 4-h exposure to staurosporine, as was seen with PARP1-GFP (Fig. S3*B*, *upper*). As PARP1 E988K-GFP retains a caspase-cleavage site, staurosporine exposure resulted in the cleavage of PARP1 E988K-GFP as was seen with PARP1-GFP (Fig. S3*B*, *lower*).

In HeLa cells, PARP1 E988K-GFP was cleaved after a 2-h exposure to staurosporine (Fig. S3*C*). Under these conditions, pull-down assay using the GST macrodomain detected the poly(ADP-ribosyl)ation of full-length and cleaved forms of PARP1 E988K-GFP (Fig. S3*C*). Pretreatment with zVAD-fmk and PJ34 suppressed staurosporine-induced poly(ADP-ribosyl)ation of PARP1 E988K-GFP. These results indicate that caspase, which is activated by staurosporine, activates endogenous PARP1, resulting in its dimerization with PARP1 E988K-GFP and transautomodification with PAR.

In PARP1 shRNA-expressing HeLa cells, staurosporine exposure resulted in the translocation of PARP1 E899K-GFP to the cytoplasm but was not accompanied by PAR production (Fig. S3*D*). Long-term exposure (8 h) to staurosporine resulted in the cleavage of both PARP1-GFP and PARP1 E988K-GFP, which were translocated to the cytoplasm (Fig. S3, *E*–*F*). Approximately 20% of their cleaved forms were localized in the cytoplasm (Fig. S3*F*). Unlike PARP1-GFP, the cleaved form of PARP1 E988K-GFP localized in the cytoplasm was not automodified by PAR and did not interact with AIF (Fig. S3, *G*–*H*). These results indicate that transautomodification of PARP1 by PAR and then its fragmentation by caspase are required for AIF binding.

### The 89-kDa PARP1 fragment also serves as a carrier for PAR translocation from the nucleus to the cytoplasm to induce AIF release in actinomycin D-induced apoptosis

**Figure 6:**
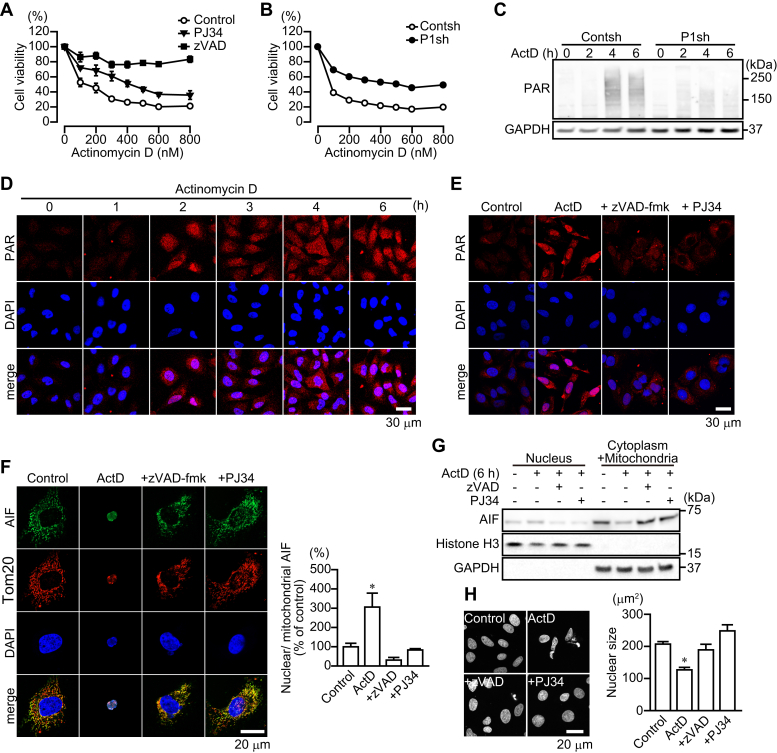
**PARP1-mediated AIF release after caspase activation after actinomycin D exposure.***A,* actinomycin D-induced cell death. HeLa cells were exposed to actinomycin D (12 h) at indicated concentrations. zVAD-fmk (50 μM) or PJ34 (10 μM) was added for 30 min before actinomycin D exposure (means ± S.E.M., n = 3). ∗∗∗*p* < 0.001 *versus* control at above 200 nM (two-way ANOVA with post hoc Tukey test). *B,* effect of PARP1 depletion on actinomycin D-induced cell death (means ± S.E.M., n = 3). HeLa cells stably expressing control or PARP1 shRNA were exposed to actinomycin D for 24 h before assessment of cell viability. ∗∗∗*p* < 0.001 *versus* control at above 100 nM (two-way ANOVA with post hoc Tukey test). *C,* time course of PAR synthesis after actinomycin D exposure. After exposure to actinomycin D (300 nM) for indicated times, HeLa cells stably expressing control and PARP1 shRNAs were subjected to Western blotting using anti-PAR antibody. GAPDH was used for a loading control. *D,* time course of PAR localization after actinomycin D exposure. After exposure to actinomycin D (300 nM) for indicated times, cells were subjected to immunocytochemistry using anti-PAR antibody (*red*). DAPI was used as a nuclear marker. *E,* effect of caspase and PARP inhibition on PAR localization. zVAD-fmk (50 μM) or PJ34 (10 μM) was added before actinomycin D (300 nM) exposure. HeLa cells were subjected to immunocytochemistry using anti-PAR antibody (*green*). DAPI was used as a nuclear marker. The scale bar represents 30 μm. *F,* AIF accumulation in nuclei of HeLa cells after 6-h exposure to actinomycin D. Cells were subjected to immunocytochemistry using anti-AIF antibody(*green*), anti-Tom20 antibody (*red*), and DAPI staining (*blue*). The scale bar represents 20 μm. Right graph shows the ratio of nuclear to mitochondrial AIF fluorescence. (means ± S.E.M., n = 3). ∗*p* < 0.05 *versus* control (one-way ANOVA with post hoc Tukey test). *G,* AIF localization in nuclei. After 6-h exposure to actinomycin D (300 nM), nuclear and cytoplasmic/mitochondrial fractions were separated. zVAD-fmk (50 μM) or PJ34 (10 μM) was added for 30 min before staurosporine exposure. Histone H3 and GAPDH were used as nuclear and cytoplasmic markers, respectively. *H,* nuclear shrinkage after 6-h exposure to actinomycin D. After exposure to actinomycin D (300 nM), DAPI was used to measure nuclear sizes in HeLa cells. The scale bar represents 20 μm. Right graph shows mean nuclear sizes (means ± S.E.M., n = 100–300 cells). ∗*p* < 0.05 *versus* control (one-way ANOVA with post hoc Tukey test). DAPI, 4',6-diamidino-2-phenylindole; PAR, poly(ADP-ribose).

We next investigated whether the 89-kDa PARP1 fragment acts as a carrier for PAR translocation from the nucleus to the cytoplasm in caspase-dependent apoptosis induced by another apoptosis inducer, actinomycin D, which inhibits DNA-dependent RNA polymerase. PJ34 and PARP1 shRNA partially suppressed actinomycin D-induced cytotoxicity. In contrast, zVAD-fmk completely blocked the cytotoxicity (Fig. 6, *A*–*B*). Actinomycin D-induced apoptosis was partially mediated through AIF-mediated cell death; in agreement, actinomycin D induced PAR synthesis in the nucleus and its translocation to the cytoplasm, AIF translocation from mitochondria to the nucleus, and nuclear shrinkage, whereas zVAD-fmk and PJ34 inhibited these effects (Fig. 6, *C*–*H*).

**Figure 7:**
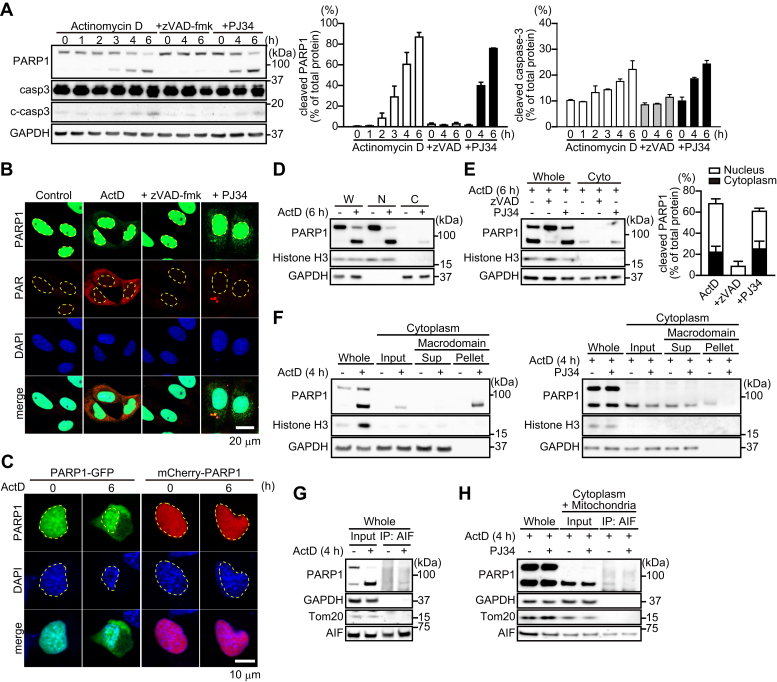
**Actinomycin D-induced PARP1 cleavage results in the translocation of 89-kDa PARP1 fragments to the cytoplasm and the binding to AIF via PAR polymers.***A,* PARP1 and caspase-3 cleavage after actinomycin D exposure. After exposure to actinomycin D (300 nM) for indicated times, cells were subjected to Western blotting with anti-PARP1 antibody and anticaspase-3 antibody. Anti-PARP1 antibody recognizes full-length PARP1 and 89-kDa PARP1 fragments. GAPDH was used as the loading control. The bar graphs represent cleaved PARP1 (*left*) and capase-3 (*right*), respectively, assessed with pooled densitometric data (means ± S.E.M., n = 3). Data are normalized to the ratio of cleaved proteins to total proteins. *B,* intracellular localization of PARP1 and PAR after 6-h exposure to actinomycin D (300 nM). Cells were subjected to immunocytochemistry using anti-PARP1 antibody (*green*), anti-PAR antibody (*red*), and DAPI staining (*blue*). *Yellow* lines indicate the position of nuclei. The scale bar represents 20 μm. *C,* localization of PARP1 constructs after 6-h exposure to actinomycin D. HeLa cells were exposed to actinomycin D (300 nM) for 6 h. Nuclei were stained with DAPI (*blue*). Yellow lines indicate the position of nuclei. The scale bar represents 10 μm. *D,* subcellular localization of PARP1 after 6-h exposure to actinomycin D (300 nM). Subcellular fractionation was performed to separate nuclei (N) and cytoplasmic (C) fractions. Histone H3 and GAPDH were used as nuclear and cytoplasmic markers, respectively. W indicates whole cells. *E,* effect of caspase and PARP inhibition on subcellular PARP1 localization. zVAD-fmk (50 μM) or PJ34 (10 μM) was added for 30 min before a 6-h exposure to actinomycin D (300 nM). The bar graph represents the ratio of cleaved PARP1 to total PARP1 in nuclear (*white*) and cytoplasmic (*black*) fractions, assessed with pooled densitometric data (means ± S.E.M., n = 3). *F,* identification of 89-kDa PARP1 fragments automodified by PAR in the cytoplasm. HeLa cells were exposed to actinomycin D (300 nM) for 4 h (*left*) without or with PJ34 (10 μM) (*right*). After subcellular fractionation, cytoplasmic fractions were subjected to GST pull-down assay using GST macrodomain. Histone H3 and GAPDH were used as nuclear and cytoplasmic markers, respectively. *G,* 89-kDa PARP1 fragments bind AIF. After 4-h exposure to actinomycin D (300 nM), cells were subjected to immunoprecipitation with anti-AIF antibody, followed by Western blotting using anti-PARP1 antibody. GAPDH was used for a loading control. *H,* 89-kDa PARP1 fragments bind AIF via PAR polymers in the cytoplasm. PJ34 (10 μM) was added for 30 min before 4-h exposure to 300 nM staurosporine. After subcellular fractionation, cytoplasmic/mitochondrial fractions were subjected to immunoprecipitation with anti-AIF antibody and then Western blotting using anti-PARP1 antibody. GAPDH and Tom20 were used as cytoplasmic and mitochondrial markers, respectively. DAPI, 4',6-diamidino-2-phenylindole; PARP1, poly(ADP-ribose) polymerase 1; PJ34, PARP inhibitor; zVAD, caspase inhibitor.

Exposure to actinomycin D resulted in PARP1 fragmentation along with caspase-3 activation (Fig. 7*A*). After PARP1 fragmentation by actinomycin D exposure, 89-kDa PARP1 fragments, but not 24-kDa PARP1 fragments, were translocated to the cytoplasm (Fig. 7*B*). Similarly, PARP1-GFP, but not mCherry-PARP1, presumably after fragmentation by caspases, was translocated to the cytoplasm (Fig. 7*C*). After 6-h exposure to actinomycin D, approximately 20% of the 89-kDa PARP1 fragment, but not full-length PARP1, was localized in the cytoplasm (Fig. 7, *D*–*E*). zVAD-fmk, but not PJ34, prevented both PARP1 fragmentation and the cytoplasmic localization of 89-kDa PARP1 fragments (Fig. 7*E*).

In the cytoplasm, 89-kDa PARP1 fragments were still modified by PAR and interacted with AIF, in a process that was inhibited by pretreatment with PJ34 (Fig. 7, *F*–*H*). These results suggested that the 89-kDa PARP1 fragment is used as a PAR carrier to induce AIF release from mitochondria in caspase-dependent apoptosis.

## Discussion

**Figure 8:**
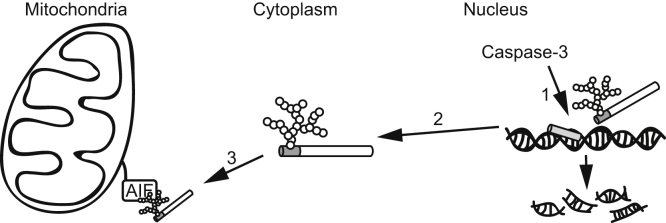
**89-kDa PARP1 fragment serves as a PAR carrier to promote AIF-mediated cell death after caspase activation.** (1) Caspase-3 activation results in the cleavage of poly(ADP-ribosyl)ated PARP1 that accumulated at DNA lesions into the 24-kDa and 89-kDa PARP1 fragments. (2) The 89-kDa PARP1 fragment with attached PAR polymers is translocated from the nucleus to the cytoplasm, whereas the 24-kDa PARP1 continuously associates with DNA lesions. (3) PAR polymers attached to the 89-kDa PARP1 fragments bind AIF, resulting in AIF release from mitochondrial membranes. (4) AIF is translocated to the nucleus where it induces large-scale DNA fragmentation by DNAase, resulting in cell death. AIF, apoptosis-inducing factor.

PARP1 fragmentation after caspase activation is well known as an early event in apoptosis (6, 8). The primary function of PARP1 is to sense and repair DNA breaks (27). PARP1 binds to DNA-strand breaks and then transfers PAR polymers to PARP1 itself, which recruits DNA repair machinery to DNA lesions (3, 5, 27). PARP1 catalyzed formation of PAR on histone, destabilizing its interactions in chromatin as a result of the negative charge of the PAR, which induces chromatin relaxation to increase the accessibility of DNA-repair machinery to DNA breaks (5, 27). Therefore, PARP1 fragmentation by caspase has been proposed to facilitate apoptosis by inhibiting these processes. In this study, we show for the first time that PARP1 fragmentation by caspase is required to generate 89-kDa PARP fragments that can be carriers of PAR to the cytoplasm to enhance AIF-mediated DNA fragmentation in a caspase-dependent process (Fig. 8).

PAR production after exposure to staurosporine and actinomycin D was suppressed by pretreatment with zVAD-fmk and PJ34, whereas caspase cleavage was not inhibited by pretreatment with PJ34, suggesting that PARP1 activated by DNA fragmentation, resulting from the activation of caspase-3–activated DNAase, might cause PAR synthesis. After 2-h exposure to staurosporine, when full-length and cleaved PARP1 coexisted in nuclei, PARP1-GFP exhibited reduced and transient association with the DNA lesion induced by laser microirradiation, compared with nonstimulated or zVAD-fmk–treated samples, whereas PARP1 D214G, a mutant form of PARP1 lacking in caspase 3 cleavage site, exhibited the same dynamics as did the nonstimulated samples. These results suggest that caspase can cleave PARP1 after PARP1 binds DNA-strand breaks and that the 89-kDa PARP1 fragment is liberated from DNA lesions because it no longer has the DNA-binding ability of the 24-kDa PARP1 fragment. Moreover, as PARP1 initiates PAR synthesis after binding DNA, PARP1-GFP could be cleaved by caspase after PAR synthesis and PAR formation on the automodification domain. In fact, we showed that the 89-kDa PARP1 fragment localized in the cytoplasm contained PAR and that its interaction with AIF required PAR. In contrast, a 2-h exposure to staurosporine resulted in more prolonged accumulation of mCherry–PARP1 bound to the DNA lesion than did nonstimulated or zVAD-fmk–treated samples. The accumulation levels, however, did not differ, suggesting that the 24-kDa PARP1 fragment is capable of DNA binding and continues to bind DNA. The continued presence of the 24-kDa PARP1 fragment prevents intact PARP1 from binding to the DNA lesion to enable DNA repair, thereby enhancing apoptosis. This finding is supported by electron micrography showing continued binding to DNA of 24-kDa PARP1 fragments generated by caspase-3 (10).

Parthanatos has been reported to participate in neuronal cell death in Parkinson's disease and after brain ischemia/reperfusion (11, 12, 13, 19, 21). However, caspase-dependent apoptosis is also seen in these diseases (28, 29, 30, 31, 32, 33). In mouse models of cerebral ischemia, PARP1 fragmentation along with caspase activation was observed and intraperitoneal administration of peptide-based caspase inhibitors including zVAD-fmk or genetic deficiency of caspase-3 protected from ischemia-induced brain injury and reduced neurological deficits (28, 29, 30, 31). Thus, cross talk between apoptosis and parthanatos pathway might have an important role in these neurological disorders.

PARP1 has been reported to be cleaved by several proteases in addition to caspase (34). Granzyme A and B are serine proteases, which are secreted together with perforin from cytotoxic T cells and natural killer cells, and are involved in eliminating cancer cells and cells infected with viruses and bacteria by inducing programmed cell death (35). Granzyme A cleaves PARP1 after K498, whereas granzyme B cleaves the same Asp-Glu-Val-Glu sequence of PARP1 as does caspase-3 (36). Granzyme B induced caspase-independent apoptosis and AIF release from mitochondria (37). Thus, caspase-independent apoptosis induced by granzyme B might generate 89-kDa PARP1 fragments that serve as PAR carriers by the same mechanism as that seen in our study.

In conclusion, we showed that PARP1 fragmentation by caspase generates an 89-kDa PARP1 fragment that serves as a PAR carrier from the nucleus to the cytoplasm to induce AIF-mediated apoptosis.

## Experimental procedures

### Antibodies

Mouse monoclonal anti-AIF (B-9) antibody and mouse monoclonal anti-Tom20 (F-10) antibody were purchased from Santa Cruz Biotechnology; rabbit polyclonal anti-PARP antibody, rabbit monoclonal GAPDH (14C10), and rabbit monoclonal Histone H3 (D1H2) antibodies from Cell Signaling Technology; mouse monoclonal anti-PARP1 (C-2-10) antibodies from Sigma-Aldrich; mouse monoclonal anti-PAR (10H) antibodies from Enzo Life Sciences; rabbit polyclonal anti-turboGFP antibody from Evrogen; and fluorescence-conjugated secondary antibodies (Alexa Fluor 488- and 555-conjugated goat anti-mouse and anti-rabbit) and horseradish peroxidase-goat anti-mouse and anti-rabbit immunoglobulin from Thermo Fisher Scientific.

### Plasmids

shRNA targeting PARP1 and GFP-PARP1 were purchased from Origene. PARP1 D214-GFP and PARP1 E988K-GFP were generated with primers (5′-TGGGAGGAGTGGATGAAGTGG-3′ and 5′-CCTCATCGCCTTTTCTCTTTCCTTC-3′) and (5′-AAGTACATTGTCTATGATATTGC-3′ and 5′-GTTATATAGTAGAGAGGTGTCATTC-3′), respectively, using KOD-plus mutagenesis kit (Toyobo). mCherry-PARP1 was gifted by Dr Negi (Doshisha Women's College of Liberal Arts).

### Cell culture

HeLa cells were incubated in Dulbecco's modified Eagle's medium (DMEM) containing 10% fetal bovine serum (FBS), 100 units of penicillin, and 100 μg/ml of streptomycin at 37 °C in a humidified atmosphere with 5% carbon dioxide. shRNA plasmid targeting PARP1 was introduced into HeLa cells using Fugene HD transfection reagent (Promega). HeLa cells were incubated with the plasmid and Fugene HD transfection reagent in Opti-MEM reduced-serum medium (Thermo Fisher Scientific). Cells stably transfected with shRNA plasmids were selectively cultured in medium with 1 μg/ml of puromycin (Thermo Fisher Scientific).

### Cell viability assay

HeLa cells (1 × 10^4^ cells) were seeded on 96-well plates and incubated for 1 day before exposure (24 h) to the indicated concentrations of staurosporine and actinomycin D (Santa Cruz Biotechnology). PARP inhibitor and caspase inhibitor were added for 30 min before staurosporine and actinomycin D exposure. Cell numbers were determined using cell counting reagent SF (Nacalai Tesque) according to the manufacturer's instructions, by measuring absorbance at 450 nm (SpectraMax M5 Microplate Reader; Molecular devices).

### Immunocytochemistry

HeLa cells (1 × 10^5^ cells), seeded on 8-well chamber plates, were incubated (1 day, 37 °C) in DMEM with 10% FBS, fixed with 4% paraformaldehyde (20 min, 4 °C), and permeabilized and blocked with 10% FBS, 1% bovine serum albumin, and 0.5% Triton X-100 (1 h, room temperature [RT]). After incubation (overnight, 4 °C) with primary antibodies, cells were treated (1 h, RT) with either Alexa-488–conjugated goat anti-rabbit IgG or Alexa-555–conjugated goat anti-mouse IgG (1:500), then washed three times with PBS, and incubated (5 min, RT) with 300 nM of 4′,6-diamidino-2-phenylindole (Thermo Fisher Scientific) to stain nuclei. No significant reactivity of cells without primary antibody was seen. Cells were imaged with a confocal microscope (Zeiss LSM 700 Meta; Carl Zeiss) equipped with an oil-immersion objective (40×, numerical aperture = 1.3). Fluorescence data were processed with ImageJ 1.37a (US National Institutes of Health).

### Live-cell imaging for microirradiation

HeLa cells (1 × 10^5^ cells), seeded on a glass-bottom dish, were transfected with PARP1 constructs (PARP1-GFP, PARP1 D214G-GFP, and mCherry-PARP1) using fugene (Promega) according to the manufacturer's instructions. After 1-day incubation, fluorescence of PARP1 constructs was observed by a confocal microscope (Zeiss LSM 700 Meta) equipped with an oil-immersion objective (63×, numerical aperture = 1.4). UV microirradiation was carried out with a 405-nm diode laser set to 100% transmission for 1 s at <1 μm in diameter within the nucleus. The microscope was equipped with a heated environmental chamber set to 37 °C. Images were taken at every 2 s for 10 min. Hoechst 33258 (10 μg/ml; Dojindo) was added to enhance DNA damage.

### Western blotting

Cells (3 × 10^5^ cells), seeded on a 6-well plate, were incubated (1 day, 37 °C) in DMEM with 10% FBS. Cell lysates were prepared with 2% SDS in 20 mM Tris-HCl (pH 7.4) containing complete protease inhibitor cocktail (Roche, Basel, Switzerland). After adjustment of protein concentration using a bicinchoninic acid kit (Thermo Fisher Scientific), cell lysates were subjected to Bis-Tris SDS-PAGE (Thermo Fisher Scientific) and then transferred to nitrocellulose membranes (Thermo Fisher Scientific). The blots were blocked with Blocking One (Nacalai Tesque) for 30 min at RT and then incubated with primary antibody. After incubation with anti-mouse and anti-rabbit IgG secondary antibodies conjugated with horseradish peroxidase, the ECL system (Fujifilm Las-3000; Fujifilm) was used for detection. The intensity of chemiluminescence was measured with ImageJ.

### Subcellular fractionation

Cells were disrupted with a homogenizer (60 strokes) in hypotonic buffer (10 mM Hepes, 10 mM potassium chloride, 1.5 mM magnesium chloride, 1 mM DTT, 0.05% nonidet P-40, and pH 7.9) containing protease inhibitor cocktail. Cell lysates were centrifuged at 400*g* for 5 min to separate the crude nuclear pellet and supernatant, which was used to generate the cytoplasmic and mitochondrial fractions. The supernatant was centrifuged at 20,000*g* for 5 min to obtain the cytoplasmic fraction. The crude nuclear pellet was resuspended in 300 μl of 0.25 M sucrose buffer (10 mM Hepes, 10 mM potassium chloride, and 1.5 mM magnesium chloride) and then mixed with 600 μl of 2.3 M sucrose buffer. The suspension was placed on 400 μl of sucrose buffer and covered by 300 μl of 0.25 M sucrose buffer. After centrifugation at 12,000*g* for 30 min, the pellet was used as the nuclear fraction.

### Preparation of GST macrodomain and GST pull-down assay

GST-fused macrodomain was expressed in *Escherichia coli* BL21 Rosetta supercompetent cells (Merck Millipore) by adding 0.5 mM IPTG at 37 °C for 6 h. GST macrodomain was extracted from competent cells in PBS by sonication with added 1% Triton X-100, with purification using Glutathione-Sepharose 4B according to the manufacturer's instructions (Cytiva) in its automodification domain as described previously (38). PARP1 automodified by PAR was incubated with recombinant human caspase-3 (10 units) in 25 μl of 50 mM Tris-HCl containing 0.1% nonidet P-40 and 5 mM DTT for 30 min at room temperature. PARP1 reaction mixture and cytoplasmic fractions (500 μg) were mixed with 20 μl of GST macrodomain immobilized on Glutathione-Sepharose 4B beads in 200 μl of hypotonic buffer containing protease inhibitor cocktail and PJ34 (10 μM; Santa Cruz Biotechnology) at 4 °C, overnight on a rotating wheel. After washing three times with hypotonic buffer, complexes were collected with lithium dodecyl sulfate sample buffer.

### Immunoprecipitation of poly(ADP-ribosyl)ated PARP1

Cells (6 × 10^5^ cells), seeded in a 6-well plate, were incubated (1 day, 37 °C) in DMEM with 10% FBS. After staurosporine or actinomycin D exposure for indicated times, cell lysates were prepared with 50 mM Tris-HCl pH 8.0, 200 mM sodium chloride, 1 mM EDTA, and 1% Triton X-100, containing protease inhibitor cocktail and 10 μM PJ34. Immunoprecipitation experiments were performed using magnetic Dynabeads covalently coupled to Protein G (Thermo Fisher Scientific) according to the manufacturer's instructions. Anti-AIF antibody (2 μg), anti-PARP1 antibody (2 μg), and anti-turboGFP antibody (1 μg) were mixed with dynabeads (50 μg) in PBS with 0.02% Tween 20 at room temperature for 1 h to bind the complexes. Cell lysates (500 μg) were incubated with antibody-labeled dynabeads at 4 °C for 1 h. After washing three times with PBS, complexes were collected with lithium dodecyl sulfate sample buffer (Thermo Fisher Scientific).

### Statistical analysis

Statistical analysis was performed using Prism (GraphPad Software). Significance was determined using Student's *t* test and one- or two-way ANOVA with post hoc Tukey test. Data are means ± S.E.M. of values from the indicated number of experiments. *p* Values <0.05 were considered significant. All representative experiments were repeated three times.

## Data availability

All representative data are contained within the article and in the supporting information.

## Conflict of interest

The authors declare that they have no conflicts of interest with the contents of this article.
