# Supplementary material for: The 89-kDa PARP1 cleavage fragment serves as a cytoplasmic PAR carrier to induce AIF-mediated apoptosis
Source: J Biol Chem. 2020 Nov 24;296:100046. doi: 10.1074/jbc.RA120.014479 (PMC7948984; doi:10.1074/jbc.RA120.014479)
Supplement: Supplementary data [file mmc1.pdf]

Supporting Information

The 89-kDa PARP1 cleavage fragment serves as a cytoplasmic PAR carrier to induce AIF-mediated apoptosis

Masato Mashimo[1*], Mayu Onishi[1], Arina Uno[1], Akari Tanimichi[1], Akari Nobeyama[1], Mana Mori[1], Sayaka Yamada[1], Shigeru Negi[2], Xiangning Bu[3#], Jiro Kato[3], Joel Moss[3], Noriko Sanada[4], Ryoichi Kizu[4], Takeshi Fujii[1]

[1]Department of Pharmacology, Faculty of Pharmaceutical Sciences, Doshisha Women's College of Liberal Arts, Kyotanabe, Kyoto, Japan

[2]Department of Clinical Pharmacy, Faculty of Pharmaceutical Sciences, Doshisha Women's College of Liberal Arts, Kyotanabe, Kyoto, Japan

[3]Pulmonary Branch, National Heart, Lung, and Blood Institute, National Institutes of Health, Bethesda, MD, USA

[4]Department of Clinical Pharmacy, Faculty of Pharmaceutical Sciences, Doshisha Women's College of Liberal Arts, Kyotanabe, Kyoto, Japan

[#]Present address: Department of Anatomy and Cell Biology
GW School of Medicine and Health Science

[*]Corresponding author: Masato Mashimo
E-mail: mmashimo@dwc.doshisha.ac.jp

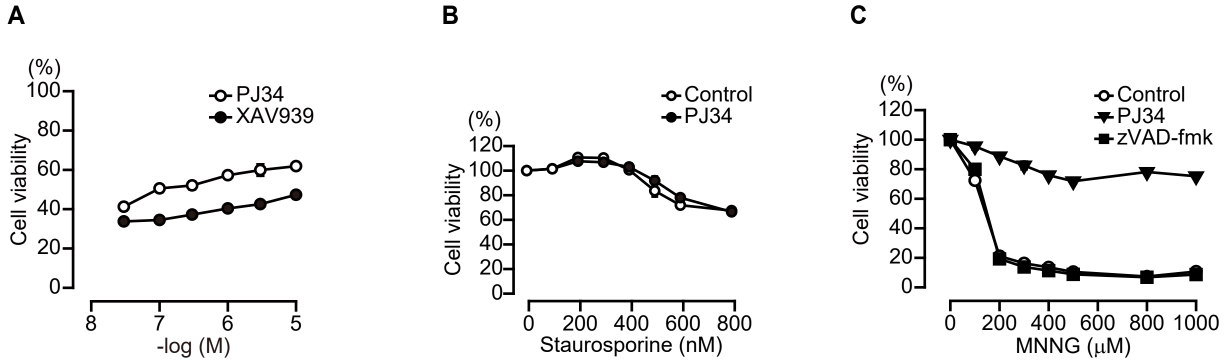

Fig. S1 Effect of PARP and caspase inhibitors on caspase-dependent and independent cell death.

A. Effect of a tankyrase inhibitor on staurosporine-induced cell death. HeLa cells were treated with PJ34 and XAV979 at the indicated concentrations for 30 min before 24-h exposure to staurosporine (300 nM). Means ± SEM (n = 3)

B. Effect of PJ34 on staurosporine-induced cell death in HeLa cells expressing PARP1 shRNA. HeLa cells were exposed to staurosporine (6 h) at the indicated concentrations. PJ34 (10 μM) was added for 30 min before staurosporine exposure. (means ± SEM, n = 3)

C. PARP-dependent cell death after MNNG exposure. HeLa cells were exposed to MNNG (12 h) at the indicated concentrations. PJ34 (10 μM) or zVAD-fmk (50 μM) was added for 30 min before MNNG exposure. (means ± SEM, n = 3) $^{***}P < 0.001$ vs. control at above 200 μM (two-way ANOVA with *post hoc* Tukey test)

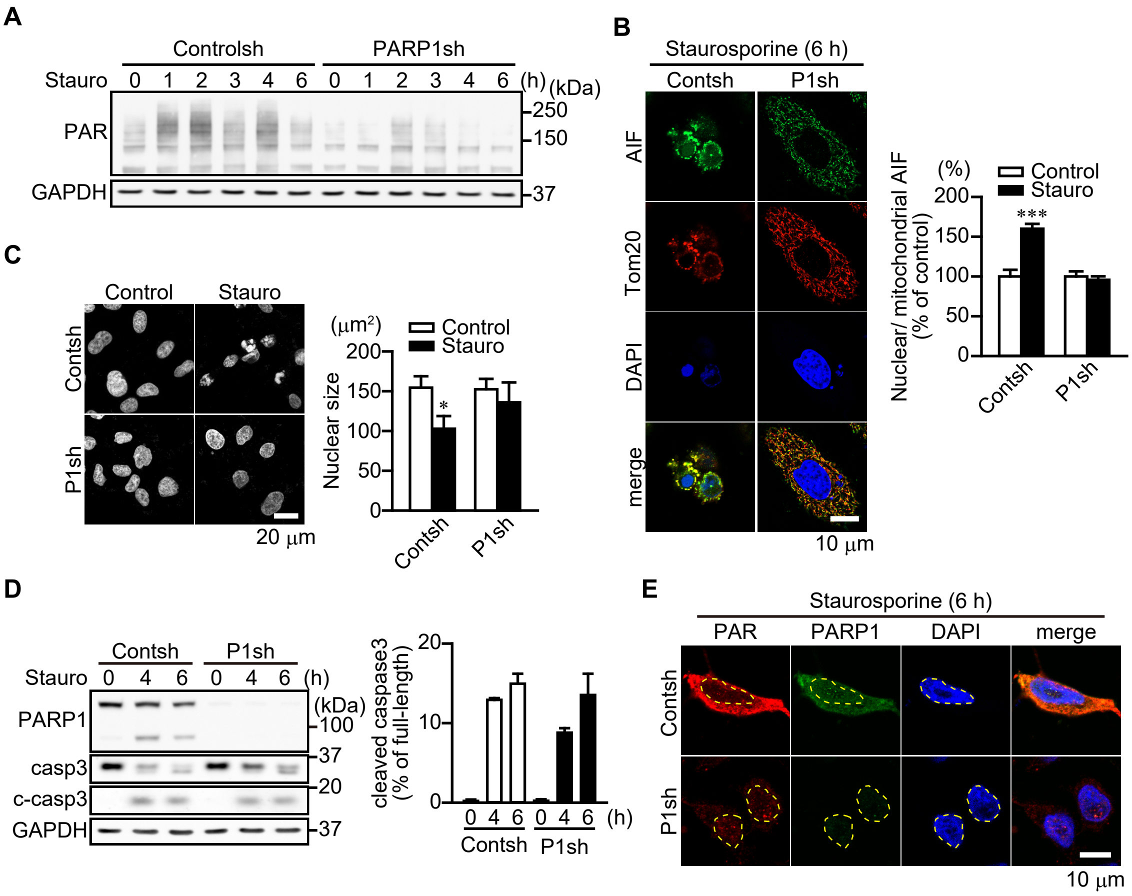

Fig. S2 PARP1-mediated PAR synthesis and AIF release after staurosporine exposure

A. PARP1-mediated PAR synthesis following incubation with 300 nM staurosporine for indicated times. HeLa cells stably expressing control or PARP1 shRNA were subjected to Western blotting using anti-PAR antibody and anti-GAPDH antibody.

B. Effect of PARP1 depletion on AIF localization. After 6-h exposure to staurosporine (300 nM), AIF localization was observed by immunocytochemistry using anti-AIF antibody. Scale bar, 10 μm. Right graph shows the ratio of nuclear to mitochondrial AIF fluorescence. (means ± SEM, n = 100-300 cells) $^{***}P < 0.001$ vs. control (two-way ANOVA with *post hoc* Tukey test)

C. Effect of PARP1 depletion on nuclear shrinkage. After 6-h exposure to staurosporine (300 nM), nuclear size was assessed by staining with DAPI. Scale bar, 20 μm. Right graph shows mean nuclear sizes. (means ± SEM, n = 100-300 cells) $^{*}P < 0.05$ vs. control (one-way ANOVA with *post hoc* Tukey test)

D. Effect of PARP1 depletion on PARP1 fragmentation and caspase-3 activation. After 4-h or 6-h exposure to staurosporine (300 nM), cells were subjected to Western blotting using anti-PARP1 antibody and anti-caspase-3 antibody. GAPDH was used as a loading control.

E. Effect of PARP1 depletion on PAR translocation to the cytoplasm. After 6-h exposure to staurosporine (300 nM), cells were subjected to immunocytochemistry using anti-PAR antibody (red) and anti-PARP1 antibody (green) and DAPI staining (blue). Scale bar, 10 μm.

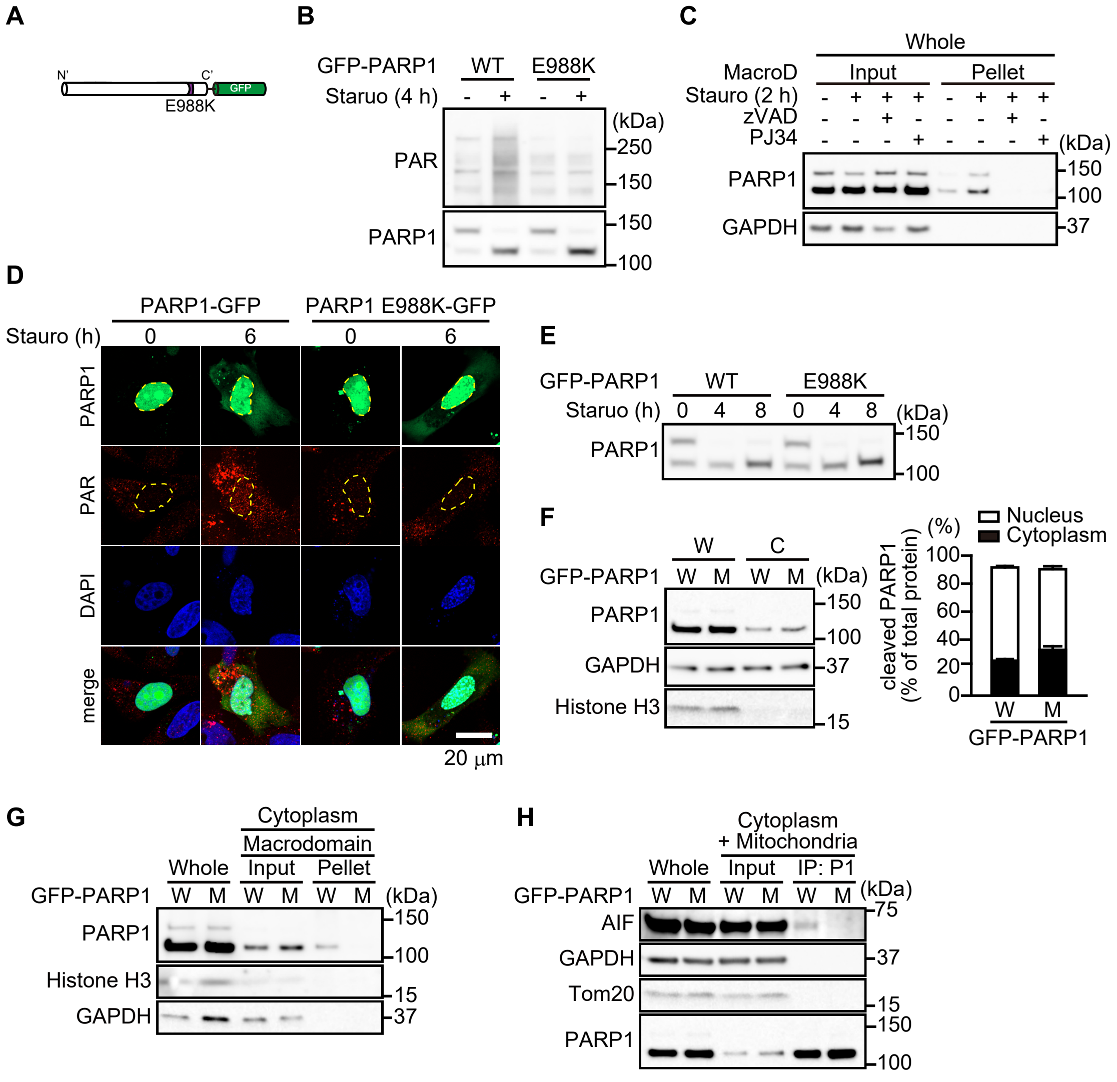

Fig. S3 Subcellular localization and poly(ADP-ribosyl)ation modification of PARP1 catalytically inactive mutant after staurosporine exposure

A. Schematic diagram of PARP1 E988K-GFP construct.

B. PAR synthesis by PARP1-GFP WT and E988K in HeLa cells expressing PARP1 shRNA. Cells were subjected to Western blotting using anti-PAR antibody and anti-PARP1 antibody.

C. Trans-automodification of PARP1 E988K-GFP after staurosporine exposure in HeLa cells. HeLa cells expressing PARP1 E988K-GFP was exposed to staurosporine (300 nM) for 2 h after treatment with zVAD-fmk (50 μM) or PJ34 (10 μM) and then subjected to GST pull-down assay using GST-macrodomain. PARP1-GFP was detected in Western blotting with anti-turboGFP antibody. GAPDH was used as a whole cell marker.

D. Localization of PARP1 E988K-GFP after 6-h exposure to staurosporine. HeLa cells were exposed to 300 nM staurosporine for 6 h. Cells were subjected to immunocytochemistry using anti-PAR antibody (red) and DAPI staining (blue). Yellow lines indicate the position of nuclei. Scale bar, 20 μm.

E. Fragmentation of PARP1 E988K-GFP following long-term exposure to staurosporine. HeLa cells expressing PARP1 shRNA with PARP1-GFP (WT) or PARP1 E988K-GFP (E988K) were exposed to staurosporine (300 nM) for indicated times. PARP1-GFP was detected by Western blotting with anti-turboGFP antibody.

F. Cytoplasmic localization of cleaved PARP1 E988K-GFP after exposure to staurosporine. HeLa cells expressing PARP1 shRNA with PARP1-GFP (W) or PARP1 E988K-GFP (M) were exposed to staurosporine (300 nM) for 8 h. PARP1-GFP was detected by Western blotting with anti-turboGFP antibody. The bar graph represents the ratio of cleaved PARP1 to total PARP1 in nuclear (white) and cytoplasmic (black) fractions, assessed with pooled densitometric data (means ± SEM, n = 3).

G. Cleaved form of PARP1 E988K-GFP is not auto-modified by PAR in the cytoplasm. HeLa cells expressing PARP1 shRNA with PARP1-GFP (W) or PARP1 E988K-GFP (M) were exposed to 300 nM staurosporine for 8 h without or with PJ34 (10 μM). After subcellular fractionation, cytoplasmic fractions were subjected to GST pull-down assay using GST-macrodomain. Histone H3 and GAPDH were used as nuclear and cytoplasmic markers, respectively.

H. Cleaved form of PARP1 E988K-GFP does not bind AIF via PAR polymers in the cytoplasm. HeLa cells expressing PARP1 shRNA with PARP1-GFP (W) or PARP1 E988K-GFP (M) were exposed to staurosporine (300 nM) for 8 h without or with PJ34 (10 μM). After subcellular fractionation, cytoplasmic/mitochondrial fractions were subjected to immunoprecipitation with anti-turboGFP antibody and then Western blotting using anti-AIF antibody. GAPDH and Tom20 were used as cytoplasmic and mitochondrial markers, respectively.
